# Empowering nanophotonic applications via artificial intelligence: pathways, progress, and prospects

**DOI:** 10.1515/nanoph-2024-0723

**Published:** 2025-02-13

**Authors:** Wei Chen, Shuya Yang, Yiming Yan, Yuan Gao, Jinfeng Zhu, Zhaogang Dong

**Affiliations:** Institute of Electromagnetics and Acoustics and Key Laboratory of Electromagnetic Wave Science and Detection Technology, Xiamen University, Xiamen, Fujian 361005, China; Quantum Innovation Centre (Q.InC), Agency for Science Technology and Research (A*STAR), 2 Fusionopolis Way, Innovis #08-03, Singapore 138634, Republic of Singapore; Science, Mathematics, and Technology (SMT), Singapore University of Technology and Design (SUTD), 8 Somapah Road, Singapore 487372, Singapore

**Keywords:** metasurface, artificial intelligence, quantum computing, machine learning, neural network

## Abstract

Empowering nanophotonic devices via artificial intelligence (AI) has revolutionized both scientific research methodologies and engineering practices, addressing critical challenges in the design and optimization of complex systems. Traditional methods for developing nanophotonic devices are often constrained by the high dimensionality of design spaces and computational inefficiencies. This review highlights how AI-driven techniques provide transformative solutions by enabling the efficient exploration of vast design spaces, optimizing intricate parameter systems, and predicting the performance of advanced nanophotonic materials and devices with high accuracy. By bridging the gap between computational complexity and practical implementation, AI accelerates the discovery of novel nanophotonic functionalities. Furthermore, we delve into emerging domains, such as diffractive neural networks and quantum machine learning, emphasizing their potential to exploit photonic properties for innovative strategies. The review also examines AI’s applications in advanced engineering areas, e.g., optical image recognition, showcasing its role in addressing complex challenges in device integration. By facilitating the development of highly efficient, compact optical devices, these AI-powered methodologies are paving the way for next-generation nanophotonic systems with enhanced functionalities and broader applications.

## 1 Introduction

**Nanophotonics** explores the interaction between light and matter at the nanometer scale, a field that has experienced significant growth driven by advancements in micro- and nanotechnology. These innovations have enabled a wide range of applications, including miniaturized optoelectronic detectors and spectrometers, high-resolution imaging systems, advanced sensing platforms, compact optical emitters, structural coloration based on iridescent nanostructures, nonlinear optical phenomena, tunable photonic components, optical holography, metasurface-enabled optically variable devices, and technologies for quantum information processing [1], [2], [3], [4], [5]. At the same time, light is inherently multidimensional, being characterized by its attributes, such as wavelength, intensity, and polarization. Simultaneous analysis of these dimensions is essential for advancing optical communication, remote sensing, and chemical and biological characterization. Additionally, it plays a pivotal role in driving the miniaturization of optical devices, enabling more compact and efficient photonic technologies [6], [7], [8], [9], [10], [11], [12], [13]. On the other hand, designing and optimizing nanophotonic devices, however, involves solving complex multiparameter problems, often requiring extensive numerical explorations [13], [14], [15], [16], [17], [18], [19], [20], [21], [22], [23]. As data volumes and design complexities continue to increase, traditional methods that rely on conventional iterations and computationally intensive processes encounter fundamental limitations. These challenges impede the efficient development of next-generation nanophotonic technologies, highlighting the need for innovative design approaches and optimization techniques.

In parallel, artificial intelligence (AI) has emerged as a transformative paradigm, driving advancements across diverse fields such as healthcare, chemistry, electronics, and manufacturing [24], [25], [26], [27], [28], [29], [30]. AI techniques, particularly deep learning, have shown immense potential in accelerating nanophotonics research. For example, AI can efficiently optimize device configurations, predict material performance, and uncover hidden physical principles that are challenging to discern using conventional approaches [31], [32]. Moreover, AI’s capacity to process large datasets and handle complex calculations offers unique opportunities to investigate the intricate interactions between light and matter at the nanoscale [33], [34].

Interestingly, the relationship between AI and nanophotonics is mutually reinforcing. Photonics, with its unique wave-based properties, has been employed to develop diffractive optical networks and other AI-inspired architectures, significantly improving computational speed and energy efficiency [35]. This synergy has catalyzed groundbreaking innovations, such as optical quantum machine learning and multifunctional on-chip systems based on metasurfaces, pushing the interdisciplinary boundaries of AI for science-driven scientific advancements [36].

**Figure 1:**
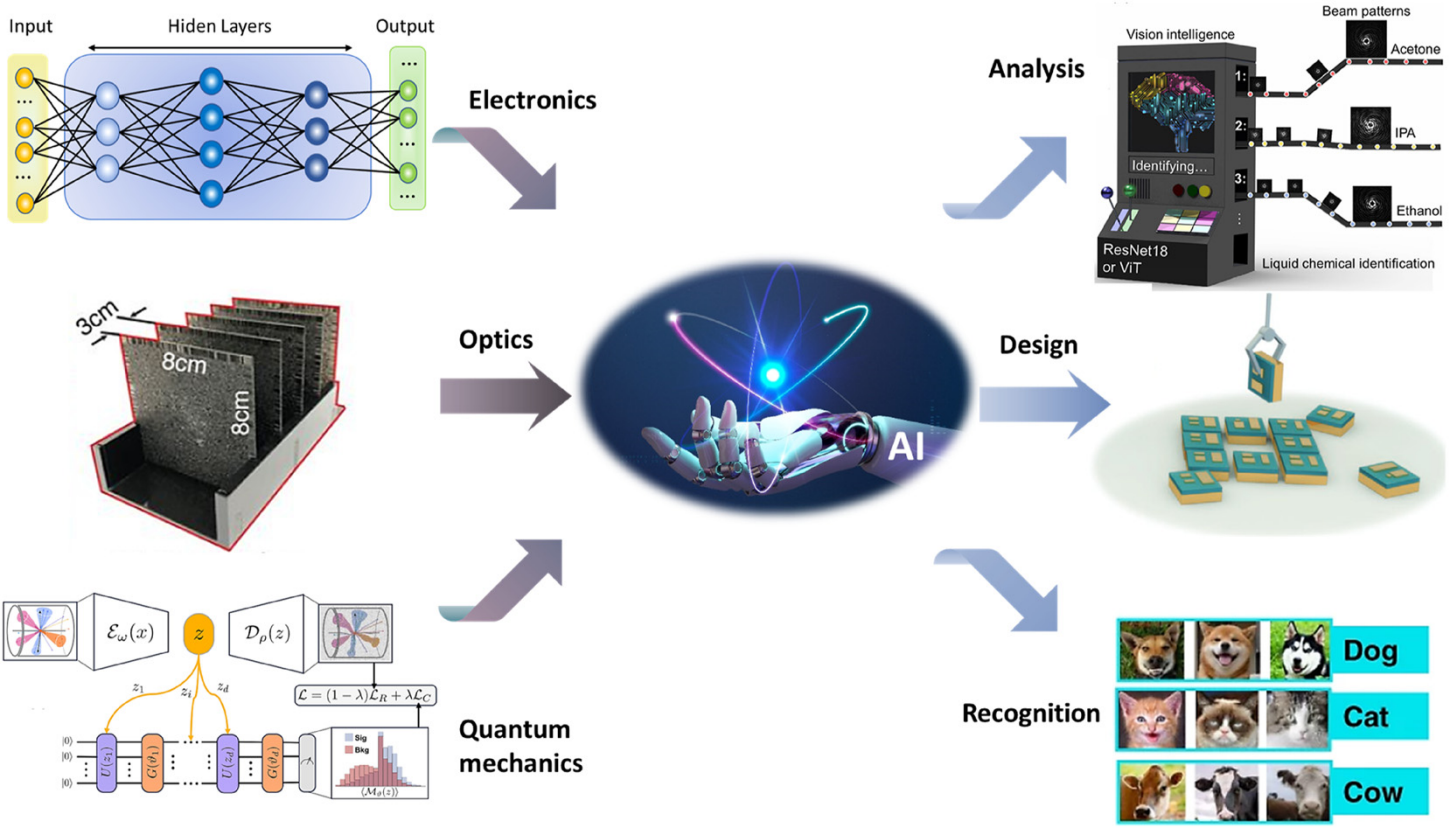
Empowering nanophotonic applications via artificial intelligence based on electronic, optical, and quantum devices. Design: adapted from Ma et al. Advanced Materials 34.16 (2022): 2110022 [30]. Copyright 2023, Wiley. Recognition: adapted from Luo et al. Light: Science & Applications 11.1 (2022): 158 [31]. Copyright 2023, Nature. Analysis: adapted from Li et al. ACS Photonics 10.3 (2023): 780–789 [32]. Copyright 2023, American Chemical Society. Quantum mechanics: Belis et al. Machine Learning: Science and Technology 5.3 (2024): 035010. [34]. Copyright 2024 Institute of Physics. Optics: Lin et al. Science 361.6406 (2018): 1004–1008 [35]. Copyright 2018, American Association for the Advancement of Science. Licensed under a Creative Commons Attribution 4.0 International License. Other parts were drawn by the authors.

This review examines the bidirectional relationship between artificial intelligence (AI) and nanophotonics, as illustrated in Figure 1. It highlights how AI-driven methodologies have revolutionized nanophotonics by optimizing device design, facilitating rapid exploration of high-dimensional parameter spaces, and predicting the behavior of complex systems with exceptional precision. Simultaneously, it explores how advancements in nanophotonics contribute to the development of AI technologies. By analyzing recent studies and breakthroughs, this review provides a comprehensive overview of the transformative interplay between these fields, paving the way for next-generation optical devices with enhanced performance and functionalities.

## 2 Fundamentals of AI for nanophotonics

AI has become a driving force in nanophotonics research, offering advanced tools for analyzing complex datasets, optimizing device designs, and exploring novel optical properties. AI methods, particularly machine learning (ML), enable researchers to tackle intricate problems that would otherwise require prohibitive computational resources. These approaches can be broadly categorized into two primary areas: neural networks and intelligent algorithms [37].

### 2.1 Neural networks

Neural networks (NNs) are pivotal in advancing the field of nanophotonics by enabling data-driven analysis, structural optimization, and performance prediction [38]. Different types of neural networks offer tailored solutions for a variety of nanophotonic challenges.

#### 2.1.1 Multilayer perceptrons (MLPs)

**Figure 2:**
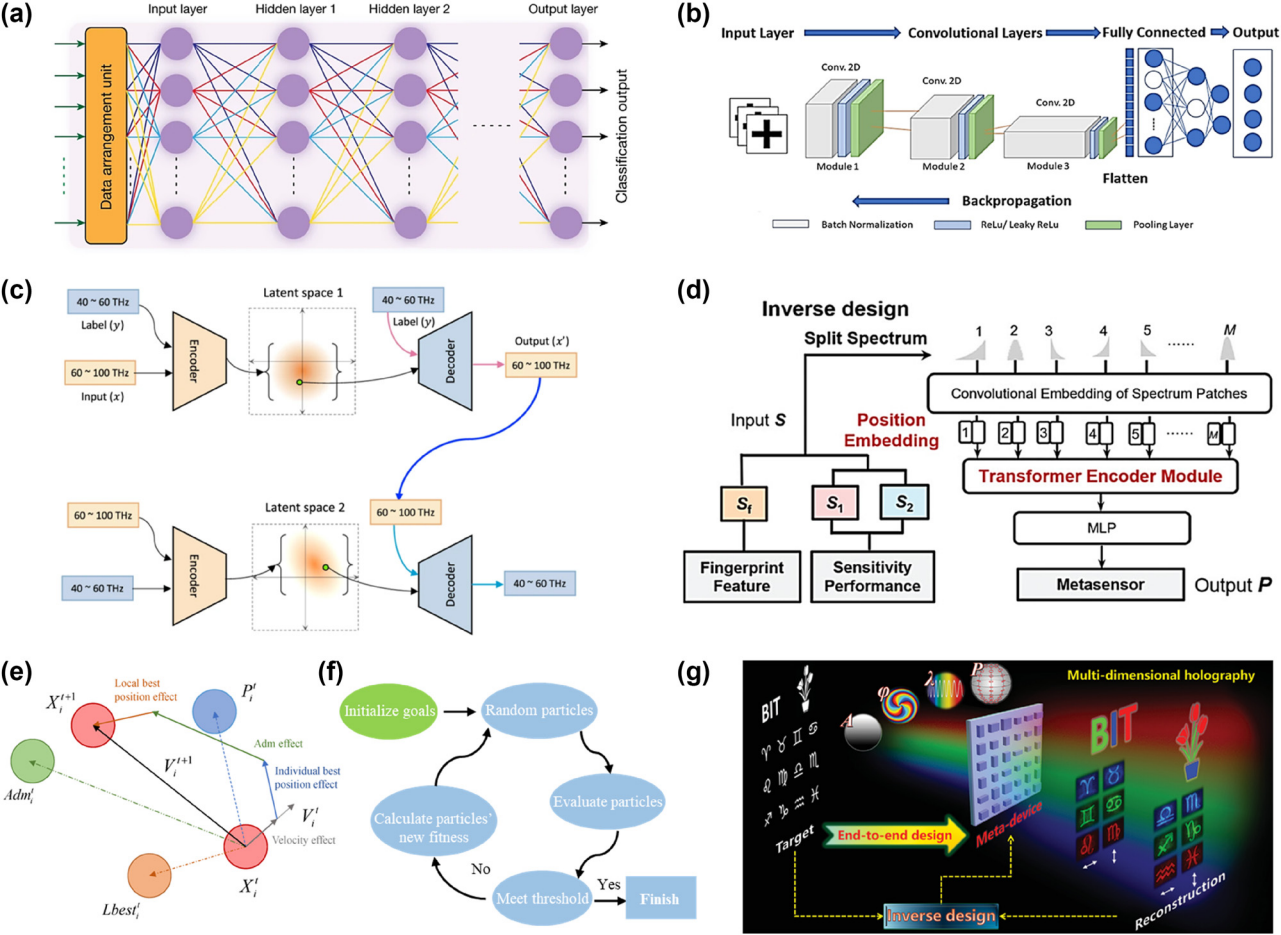
Fundamentals of AI for nanophotonics, illustrating key neural network frameworks and intelligent algorithm flowcharts. Schematics of (a) multilayer perceptron (MLP) architecture. Adapted from Ashtiani et al. Nature 606.7914 (2022): 501–506 [39]. Copyright 2022, Nature. (b) Convolutional neural network (CNN). Adapted from Razi et al. Materials & Design 236 (2023): 112475 [41]. Copyright 2023, Elsevier. (c) Variational autoencoder (VAE). Adapted from Chen et al. Nature Communications 14.1 (2023): 4872 [42]. Copyright 2023, Nature. (d) Transformer model. Adapted from Gao et al. Advanced Science (2024): 2405750 [43]. Copyright 2024, Wiley. (e) Particle swarm optimization (PSO) principle. Adapted from Yuan et al. Processes 11.1 (2022): 26 [44]. Copyright 2022, Multidisciplinary Digital Publishing Institute, and (f) PSO flow diagrams. Adapted from Liu et al. IEEE Access 9 (2021): 92941–92951 [45]. Copyright 2021, The Institute of Electrical and Electronics Engineers. (g) End-to-end inverse design algorithm. Adapted from Yin, et al. Advanced Materials (2024): 2312303 [46]. Copyright 2021, The Institute of Electrical and Electronics Engineers.

Multilayer perceptrons (MLPs) are fundamental feedforward neural networks suitable for tasks involving low-dimensional or linear datasets, such as material property prediction and basic parameter optimization. An MLP model typically consists of three main layers: an input layer, one or more hidden layers, and an output layer (as depicted in Figure 2a) [39]. The input layer processes feature extracted from the dataset, while the hidden layers perform computations using weighted connections and nonlinear activation functions like the Rectified Linear Unit (ReLU) [40]. Although MLPs are well-suited for straightforward analytical tasks, they encounter difficulties on handling data with high dimensionality or complexity. Figure 2a illustrates the structure of a deep neural network, where input data are first preprocessed through the input neurons, followed by intermediate (hidden) layers. Finally, the classification outcome is generated by the output neurons. Each neuron calculates its output by applying a nonlinear activation function to the weighted sum of its inputs.

#### 2.1.2 Transformer models

The introduction of Transformer models by Vaswani et al. in 2017 marked a significant advancement in the field of natural language processing (NLP), as these models employed self-attention mechanisms that superseded conventional sequence models. [47]. These mechanisms excel at capturing long-range dependencies in input sequences, enhancing generative tasks like text completion and translation. Generative Pretrained Transformer (GPT) models, derived from this architecture, have further expanded capabilities [48]. In nanophotonics, Transformer model is adapted with dimensionality reduction techniques to address mismatches between input and output dimensions (Figure 2b) [43]. The input spectrum is segmented into patches to address the significant dimensional mismatch between the input ***S*** and output ***P***. Each patch undergoes convolution for feature extraction, followed by positional encoding, producing a sequence of vectors. These vectors are then fed into the Transformer encoder module, which is connected to an MLP layer, ultimately yielding the predicted metastructure parameters. The encoder module comprises *L* identical layers, each containing a multi-head attention mechanism and a feed-forward MLP. Within each attention head, the input sequence is transformed using three learnable weight matrices into the query, key, and value vectors (*Q*, *K*, *V*). The self-attention mechanism computes attention scores as follows [43]:

(1) $$\text{Attention}\left(Q,K,V\right)=\text{Softmax}\left(\frac{QK^{T}}{\sqrt{d_{k}}}\right)V$$

here *d*_*k*_ is the dimension of *Q* and *K*. The dot products of query with all the keys are normalized and weighted using the Softmax function. These attention weights are then applied to the values *V*. The outputs of all attention heads are concatenated as [43]:

(2) $$\text{MultiHead}\left(Q,K,V\right)=\text{Concat}\left(\text{Hea}{\text{d}}_{1},\ldots \text{Hea}{\text{d}}_{i},\ldots ,\text{Hea}{\text{d}}_{Z}\right)W^{\text{O}}$$

where *Z* denotes the number of heads, Head_*i*_ is the output of the *i*th head, and *W* ^O^ is the projection matrix for all heads. The result of the multi-head attention mechanism is subsequently passed to the MLP layer for final processing.

#### 2.1.3 Variational autoencoders (VAEs)

Variational autoencoders (VAEs) are generative models capable of producing new data samples resembling the training data [42]. In nanophotonics, VAEs are particularly effective for inverse design tasks requiring specific optical properties. By learning data distributions, VAEs generate unconventional designs that optimize light–matter interactions, light propagation control, and device performance. For the VAE architecture, as shown in Figure 2c, only the generation network is illustrated for simplicity, while the elimination network can be constructed by switching low and high frequencies. Reflection coefficients in the high-frequency band (60–100 THz) are discretized into 668 data points. Due to the symmetry of the elliptical pattern, only the three coefficients (*R*_*xx*_, *R*_*xy*_, *R*_*yy*_) are utilized, forming a 2004-dimensional input/output vector. The network’s operation involves reconstructing the input *x* as the output *x*′, aligning with the principles of autoencoders and VAEs. Labels, discretized from 40 to 60 THz into 333 data points, transform the unsupervised VAE model into a supervised conditional VAE by providing a supervised training target.

#### 2.1.4 Convolutional neural networks (CNNs)

Convolutional neural networks (CNNs) excel at analyzing spectral and microscopy images due to their advanced image processing capabilities. By extracting spatial features through convolutional layers, CNNs enable the identification of nanostructure characteristics, the detection of material defects, and the optimization of structural parameters. A 2D-CNN model for metasurface prediction is depicted in Figure 2d [41]. The mean-square error and accuracy are calculated by the following expressions [41]:

(3) $$\text{MES}=\frac{1}{N}\overset{N}{\underset{i=1}{\sum }}{\left(y_{i}-{\hat{y}}_{i}\right)}^{2}$$

(4) $$\text{Accuracy}=\frac{\text{Number}\;\;\text{of}\;\;\text{correct}\;\;\text{predictions}}{\text{Total}\;\;\text{number}\;\;\text{of}\;\;\text{predictions}}$$

where *N* is the number of data points, *y*_*i*_ is the trained value, and ${\hat{y}}_{i}$ denotes the validated value. The CNN model’s performance is evaluated by comparing the predicted spectrum against simulation results using the mean absolute accuracy. Other networks, such as ResNet and recurrent neural networks (RNNs), also contribute to nanophotonics research, although they are not expanded upon here [49], [50], [51], [52], [53].

### 2.2 Intelligent algorithms

In addition to neural networks, traditional AI algorithms play a crucial role in supporting nanophotonic research by enhancing data analysis and optimization processes.

#### 2.2.1 Classification algorithms

Classification algorithms such as Random Forests, Support Vector Machines (SVM), and K-means clustering play essential roles in identifying and categorizing nanophotonic materials and structures. For instance, SVMs classify material phases based on spectral responses, while Random Forests detect structural defects in nanostructures [54].

#### 2.2.2 Filtering algorithms

Filtering algorithms are crucial for preprocessing experimental data and images, removing noise, and extracting relevant features. Common techniques include Kalman filters, low-pass filters, and high-pass filters. Kalman filters correct noisy measurements, low-pass filters eliminate high-frequency noise, and high-pass filters enhance edges in microscopy images, revealing fine structural details [55].

#### 2.2.3 Optimization algorithms

Optimization algorithms are indispensable for structural design and parameter tuning. Methods such as genetic algorithms, Bayesian optimization, and particle swarm optimization (PSO) efficiently navigate parameter spaces in multiobjective optimization tasks. Notably, adjoint optimization is widely used in nanophotonics and metasurface design to optimize complex structures and meet specific optical performance requirements, such as high-efficiency lenses, filters, or waveguides [56], [57]. In scenarios where multiple performance metrics need to be optimized simultaneously, adjoint optimization significantly reduces computational costs through its efficient gradient computation. Moreover, the integration of adjoint optimization algorithms with artificial intelligence techniques, such as neural networks, can further enhance the efficiency of photonic device design. The principle and flow of PSO mimics swarm behavior to find optimal configurations is plotted in Figure 2e and f [44], [45]. The process iterates through 30 generations, with 20 parameters evaluated per generation. Calculated and optimized values are input into a fitness function. The optimization concludes when either the maximum iterations are reached or the desired average absorption is achieved. Otherwise, particle positions are updated based on the fitness function, and the process repeats until the stopping criterion is satisfied. These techniques enable rapid identification of designs, which meets diverse performance criteria and enables on-demand design (Figure 2f) [46], [58], [59]. In order to achieve on-demand functionality, the loss function’s derivative with respect to the phase response of each meta-atom at the object plane can be expressed as [46]:

(5) $$\begin{array}{ll} \frac{\partial L}{\partial \varphi_{p,q}} & =\frac{\partial \left(\overset{M}{\underset{j=1}{\sum }}\overset{M}{\underset{i=1}{\sum }}{\left(E_{ij}E_{i,j}^{*}-I_{i,j}\right)}^{2}\right)}{\partial \varphi_{p,q}}\\ & =4\overset{M}{\underset{j=1}{\sum }}\overset{M}{\underset{i=1}{\sum }}\left[\left(E_{i,j}E_{i,j}^{*}-I_{i,j}\right)Re\left\{E_{i,j}^{*}\frac{\partial E_{i,j}}{\partial \varphi_{p,q}}\right\}\right] \end{array}$$

where $Re\left\{\ldots \right\}$ denotes the real part of the value in the bracket. Here, *i*, *j* and *p*, *q* represent the positions of each pixel or meta-atom in the image plane and object plane, respectively. The scattered field *T*_*p*,*q*_ from metasurface is represented as $A_{p,q}\exp\left(i\varphi_{p,q}\right)$ , where *A* represents the amplitude and *φ* is the phase. The Wirtinger calculus is applied for derivatives. Using the chain rule, the relationship between the loss function and the geometric size of the meta-atom (e.g., length *L*_*i*,*j*_) can be built by the following derivative [46]:

(6) $$\begin{array}{ll} \frac{\partial L}{\partial L_{p,q}} & =\frac{\partial L}{\partial \varphi_{p,q}}\frac{\partial \varphi_{p,q}}{\partial L_{p,q}}+\frac{\partial L}{\partial A_{p,q}}\frac{\partial A_{p,q}}{\partial L_{p,q}}\\ & =4Re\left\{\overset{M}{\underset{j=1}{\sum }}\overset{M}{\underset{i=1}{\sum }}\left[\left(E_{i,j}E_{i,j}^{*}-I_{i,j}\right)E_{i,j}^{*}H_{i,j,p,q}\right]\right.\\ & \;\left.\times \frac{\partial T_{p,q}}{\partial L_{p,q}}\right\} \end{array}$$

## 3 Smart design driven by machine learning

**Figure 3:**
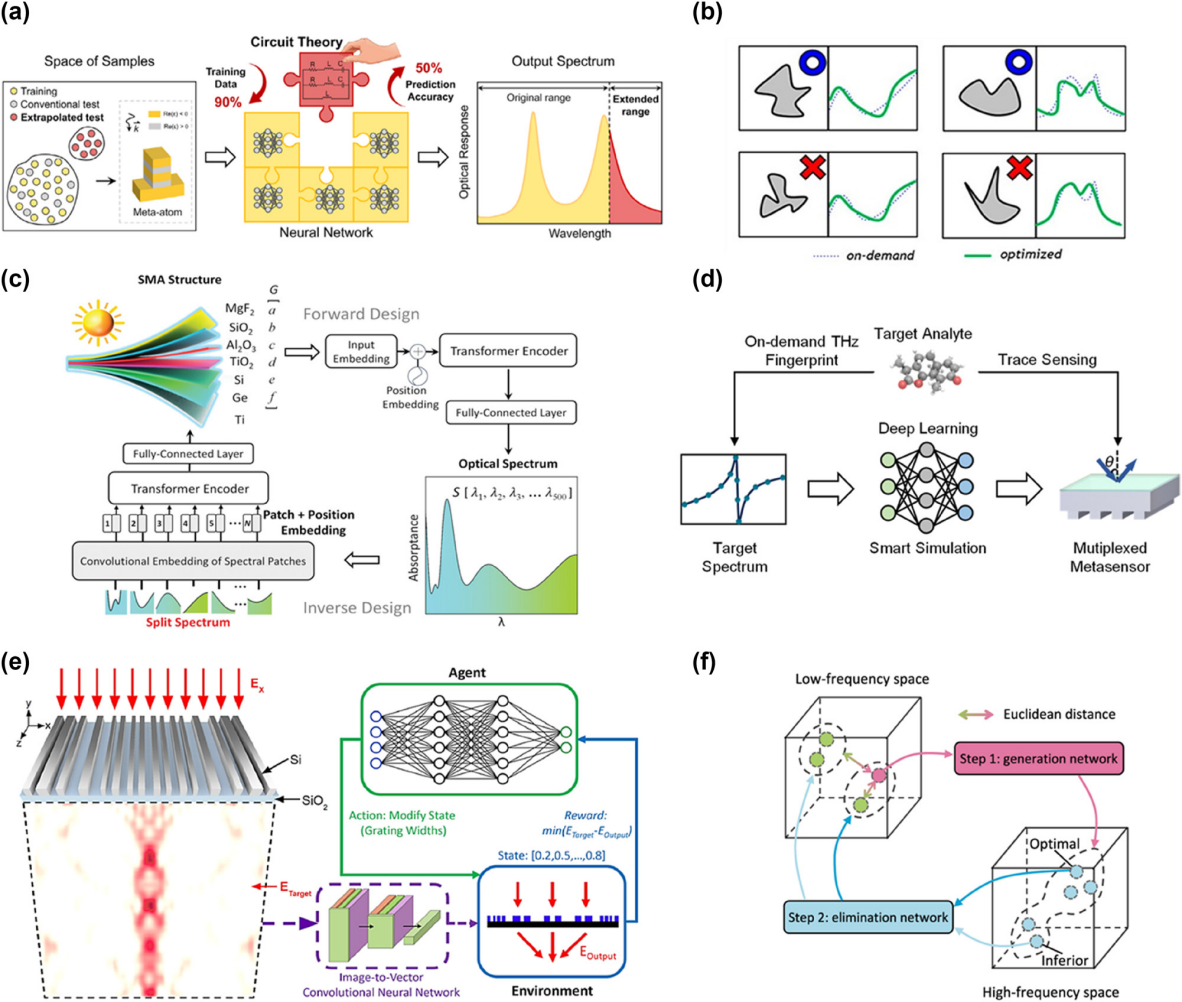
Smart design driven by machine learning. (a) Intelligent design of metamaterials by circuit -physics-driven deep learning. Adapted from Yan et al. Laser & Photonics Reviews: 2400724 [60]. Copyright 2024, Wiley. (b) Forward and inverse design of manufacturable free-form dielectric metasurfaces. Adapted from Tanrioveret et al. ACS Photonics 10.4 (2022): 875–883 [61]. Copyright 2022, American Chemical Society. (c) Schematic drawing of MST architecture for the smart design of solar metamaterial absorbers. Adapted from Chen et al. Advanced Science 10.13 (2023): 2206718 [62]. Copyright 2023, Wiley. (d) Rapid on-demand design for molecular fingerprint sensing. Adapted from Liu et al. ACS Photonics, 2024, 11(11): 4838–4845 [63]. Copyright 2024, American Chemical Society. (e) Inverse design of transmissive metagratings based on hybrid SL and RL. Adapted from Yeung et al. Optics Express 32.6 (2024): 9920–9930 [64]. Copyright 2024, The Optical Society of America. (f) The interconnection between two networks (a generation network and an elimination network). Adapted from Chen et al. Nature Communications 14.1 (2023): 4872 [42]. Copyright 2023, Nature.

The integration of AI, particularly machine learning (ML), has revolutionized the exploration of complex parameter spaces and the prediction of unknown features in nanophotonics. High-effective design methods have accelerated design processes, enabling the development of advanced photonic devices. Yan et al. proposed the circuit-theory-informed neural network (CTINN), which integrates equivalent circuit theories into deep learning (DL) (Figure 3a) [60]. CTINN not only predicts spectra with high accuracy beyond the structure’s training span but also extrapolates optical responses across extended wavelength ranges. With its physics-guided design, CTINN demonstrates superior generalization, requiring only 10 % of the training data compared to conventional models while reducing test loss by over 50 %. However, challenges such as low manufacturing feasibility, limited design freedom, and insufficient model generalizability persist.

Addressing these issues, Ibrahim-Tanriover et al. proposed a comprehensive framework for generative modeling and inverse design of manufacturable freeform metasurfaces (Figure 3b) [61]. Their approach incorporates meta-atom parameters – including cross-section shape, periodicity, refractive index, and height, into the forward network, achieving inverse optimization under manufacturing constraints. DL models have also facilitated the rapid design of high-performance broadband solar metamaterial absorbers (SMAs). Chen et al. developed a metamaterial spectrum transformer (MST) network, based on a spectrum-splitting scheme, to meet user-defined spectral requirements with higher accuracy than traditional multilayer perceptron (MLP) networks (Figure 3c) [62]. In the proposed Transformer-based deep learning framework, both forward and inverse design processes are incorporated. For the forward design, the input comprises a vector ***G*** representing the GRI-based metamaterial, where the variables *a*, *b*, *c*, *d*, *e*, and *f* denote the thicknesses of individual GRI layers. Positional embeddings are applied to enhance the representation of the input data. The input is then passed through an encoder composed of multiple identical layers, each containing two core components: multi-head attention and a position-wise feed-forward network. A fully connected layer follows the transformer encoder, enabling the generation of the predicted spectra. For the inverse design, the input is a spectral vector ***S*** covering 500 wavelength points within the range from 300 nm to 2,500 nm. These spectral data are divided into 25 patches, and each patch undergoes one-dimensional convolutional embedding. This step transforms the spectral patches into vector representations suitable for processing by the MST model. Positional information is incorporated into the sequence through positional embeddings to maintain the order of the spectral data. Ultimately, the predicted structural parameters are obtained using a Transformer Encoder, coupled with a fully connected layer, in a manner similar to the forward design process.

Liu et al. introduced a bidirectional neural network for customizing inverted all-dielectric metagratings, applied to trace THz fingerprint sensing [63]. As shown in Figure 3d, their forward network employs a divide-and-conquer strategy, using multiple subnetworks for segmental spectral prediction. This approach significantly enhances prediction accuracy compared to traditional methods. Beyond purely data-driven approaches, physics-informed AI models are gaining prominence. To enhance design performance, Yeung et al. combined reinforcement learning (RL) with supervised learning (SL) to optimize nanophotonic structures (Figure 3e) [64]. Using a convolutional neural network (CNN) for the initial inverse design of a silicon-on-insulator metagrating, they refined device performance through an RL process. This hybrid approach overcomes the limitations of individual DL methods, offering a more practical and efficient solution for nanophotonic design. In addition to forward and inverse designs, innovative methods like spectra-to-spectra design are being explored. Chen et al. proposed a generation-elimination framework for inferring optical responses from existing spectral data (Figure 3f) [42]. The generative network produces a wide range of candidate solutions, while the elimination network identifies the optimal designs. This framework, comprising an encoder, latent space, and decoder, is extendable to other photonic design fields, offering a robust tool for tackling complex challenges.

## 4 Diffractive optical networks

**Figure 4:**
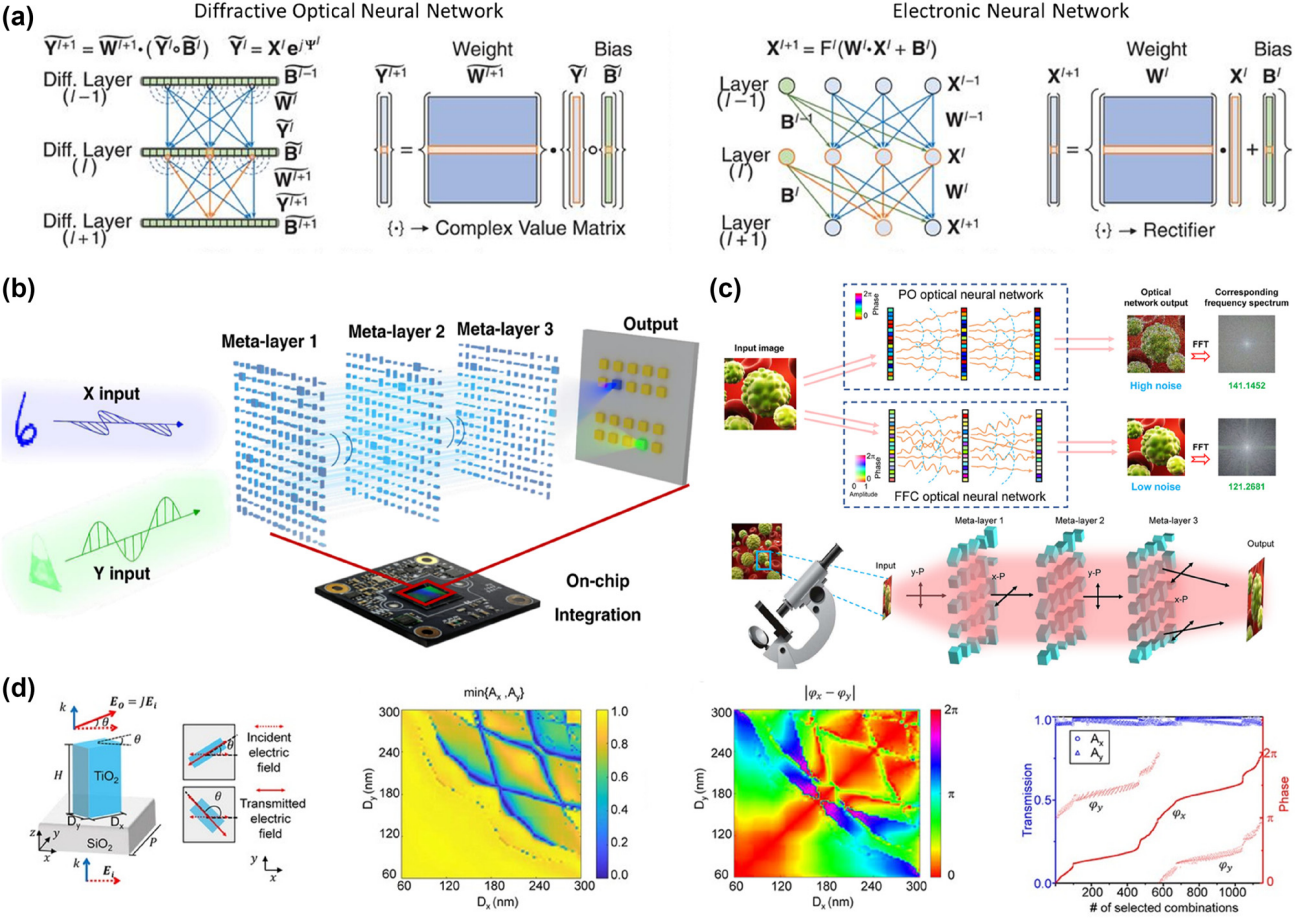
Working principle of diffractive optical networks. (a) Comparison between a D2NN and a conventional neural network. Adapted from Lin et al. Science 361.6406 (2018): 1004–1008 [35]. Copyright 2018, American Association for the Advancement of Science. (b) Optical layout of polarization-dependent object classification for the metasurface-enabled D2NN concept. Adapted from Luo et al. Light: Science & Applications 11.1 (2022): 158 [31]. Copyright 2022, Nature. (c) Schematic and framework of the full-Fourier-component optical neural meta-transformer. Adapted from Luo et al. Laser & Photonics Reviews 17.12 (2023): 2300272 [71]. Copyright 2023, Wiley. (d) Meta-units with arbitrary and independent control of amplitude and phase for D2NN. Adapted from Luo et al. Laser & Photonics Reviews 17.12 (2023): 2300272 [71]. Copyright 2023, Wiley.

Deep learning has significantly enhanced the ability to leverage computers for complex reasoning tasks. Traditional deep learning methods rely on multilayer artificial neural networks (ANNs) to learn data representations and abstract features, with successful applications across various domains, including language translation, speech recognition, medical image analysis, and image classification. However, their dependence on electronic computation imposes limitations regarding both speed and energy efficiency [65]. These limitations have prompted researchers to explore novel computational paradigms, such as all-optical machine learning frameworks like the diffractive deep neural network (D2NN), to bypass the bottlenecks of electronic computation and enable faster, more energy-efficient processing [66]. The D2NN framework represents an innovative all-optical approach to deep learning, as shown in Figure 4a. It leverages a multilayer architecture of diffractive surfaces that collectively function as a physical neural network, performing computations through optical interactions rather than digital operations [67], [68], [69], [70]. Utilizing principles of optical diffraction, the D2NN is capable of executing complex tasks at the speed of light without the need for active electrical components. Key distinctions between the D2NN and a conventional neural network:

(1) Framework: The D2NN processes data via coherent light waves, handling complex-valued inputs and incorporating multiplicative biases. The network’s weights are defined by free-space diffraction, with secondary wave interference modulated in phase and/or amplitude by each preceding layer.
(2) Speed: The D2NN performs its learned functions at light speed, using optical diffraction and passive components, whereas conventional neural networks are limited by slower electronic computation.
(3) Energy Efficiency: Since the D2NN operates through passive optical layers, it requires no additional power for computation, achieving high energy efficiency.

The optical layout of the metasurface-enabled D2NN is illustrated in Figure 4b, where light carrying information about the object is polarized in either the *x*- or *y*-direction to differentiate object types [31]. The hidden layers of the metasurface-enabled D2NN consist of polarization-multiplexed metasurfaces acting as neurons, manipulating the phase of incoming light based on its polarization. These metasurfaces direct the diffracted light to specific regions on a complementary metal-oxide-semiconductor (CMOS) chip, which serves as the network’s output layer. The training follows principles similar to conventional electronic neural networks, with input, hidden, and output layers. Using deep learning and error backpropagation, the phase distributions within each metasurface layer are iteratively optimized, converging the light energy from different channels onto distinct detection regions on the CMOS, each representing a classification [72], [73], [74], [75], [76]. This approach enables the metasurface-enabled D2NN to classify objects by their polarization-dependent optical signatures, allowing simultaneous recognition of diverse object types.

Figure 4c explains the principles of the D2NN, which is comprised of an input layer, one or more hidden transmission layers, and an output layer. Every point in the hidden layers represents a frequency and field-controlled (FFC) meta-neuron capable of modulating optical signals. Light entering the system is polarized along the *x*- or *y*-axis, providing distinct paths for information propagation based on polarization states. The training flow of the FFC optical neural meta-transformer uses the ReLU function to constrain neuron amplitude values and the sigmoid function to control phase values, with components labeled as CF (complex field), FP (free-space propagation), and BP (backpropagation) [77], [78], [79], [80]. Key processes include element-wise multiplication, cross-entropy error (CEE), and mean square error (MSE), all used to optimize neuron response for effective optical processing. Figure 4d illustrates the geometry and functional modulation of TiO_2_ meta-atoms. Meta-atoms’ physical picture can be manipulated by using the optical rotation effect to adjust the amplitude, with the help of an auxiliary polarizer acting as an isolator, and using the phase delay of birefringent structures to regulate the phase. We can employ a symmetric, unitary Jones matrix to represent the transmission matrix of the nanopillar [71],

(7) $$J_{\text{meta}}=\times \left[\begin{array}{cc} {\left(\cos\theta \right)}^{2}A_{x}{\text{e}}^{\text{i}\varphi_{x}}+{\left(\sin\theta \right)}^{2}A_{\gamma }{\text{e}}^{\text{i}\varphi_{y}} & \frac{1}{2}\text{sin}\;2\theta \left(A_{x}{\text{e}}^{\text{i}\varphi_{x}}-A_{\gamma }{\text{e}}^{\text{i}\varphi_{y}}\right)\\ \frac{1}{2}\text{sin}\;2\theta \left(A_{x}{\text{e}}^{\text{i}\varphi_{x}}-A_{\gamma }{\text{e}}^{\text{i}\varphi_{y}}\right) & {\left(\sin\theta \right)}^{2}A_{x}{\text{e}}^{\text{i}\varphi_{x}}-{\left(\cos\theta \right)}^{2}A_{\gamma }{\text{e}}^{\text{i}\varphi_{y}} \end{array}\right]$$

here (*A*_*x*_, *A*_*y*_) and (*φ*_*x*_, *φ*_*y*_) are the outgoing amplitude and phase along the *x*-axis and the orthogonal *y*-axis at *θ* = 0, respectively. After introducing the basic principles of D2NN, we next review their various disruptive applications in nanophotonics.

**Figure 5:**
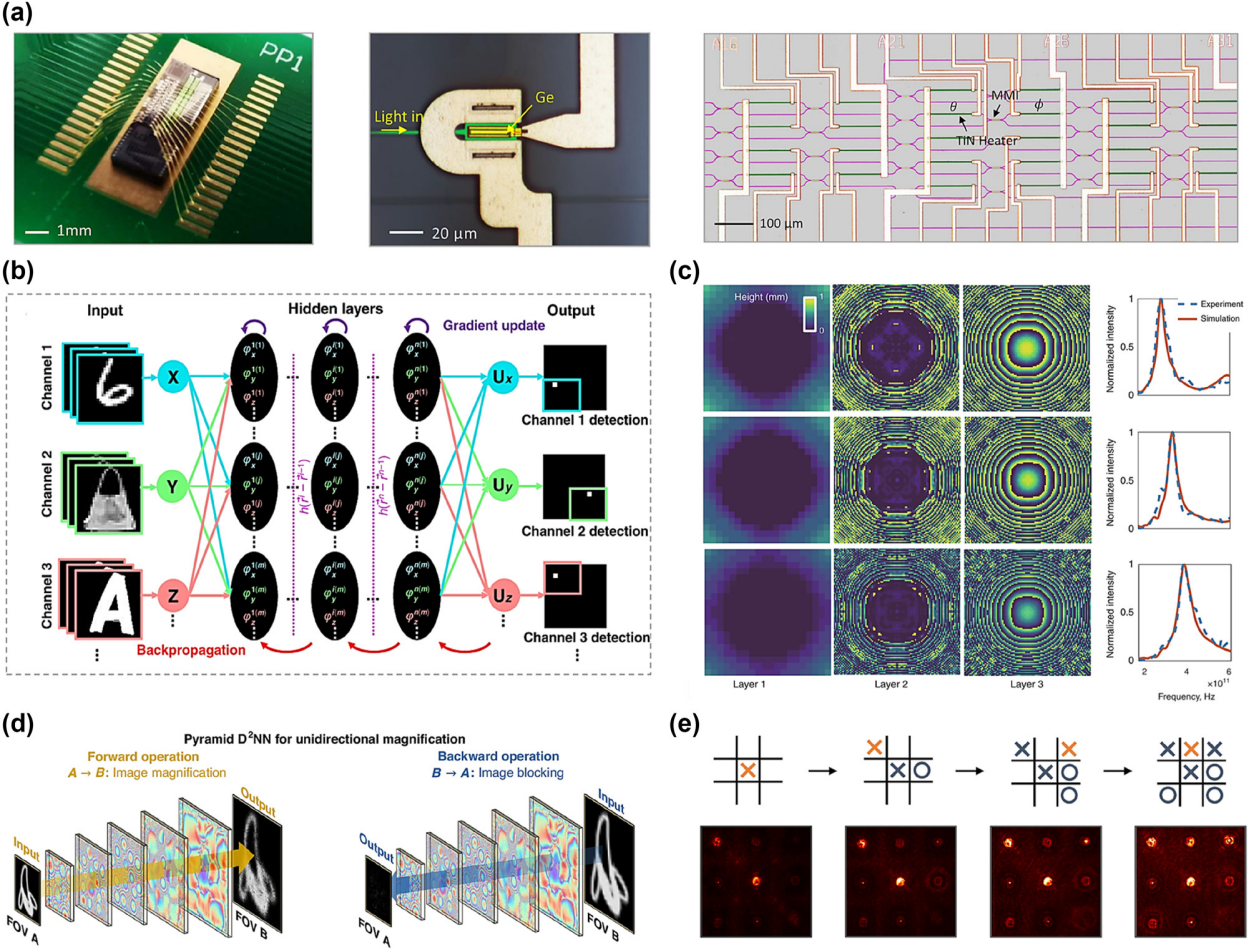
Various applications of diffractive optical networks. (a) Chip packaging and a false-color micrograph of the D2NN with integrated heaters. Adapted from Zhang et al. Nature Communications 12.1 (2021): 457 [84]. Copyright, 2021, Nature. (b) Architecture of the metasurface-enabled D2NN. The meta-units of the multiple networks are trained separately to achieve multiplexed phase distributions, optimized using an error backpropagation algorithm executed on a computer. Adapted from Luo et al. Light: Science & Applications 11.1 (2022): 158 [31]. Copyright 2023, Nature. (c) Optimized and learned thickness profiles of three diffractive layers along with the corresponding simulated (red) and experimentally measured (dashed blue) spectral responses. Adapted from Luo et al. Light: Science & Applications 8.1 (2019): 112 [88]. Copyright, 2019, Springer Nature. (d) Pyramid diffractive optical networks for unidirectional image magnification. The diffractive network enhances images in the forward networks and effectively blocks in the inverse networks. Adapted from Bai et al. Light: Science & Applications 13.1 (2024): 178 [89]. Copyright, 2024, Nature. (e) The sequential control of the all-optical D2NNs in playing the classic tic-tac-toe games. Adapted from Qiu et al. Advanced Photonics Nexus 3.4 (2024): 046003 [90]. Copyright 2022, Society of Photo-Optical Instrumentation Engineers.

Traditional digital electronic computing platforms cannot perform true complex-valued representations and operations [81], [82], [83]. Zhang et al. implemented a true complex-valued D2NN on a single optical neural chip (ONC), as shown in Figure 5a [84]. They benchmarked the performance of their complex-valued ONC across four different scenarios: a basic Boolean task, classifying species in an iris dataset, classification of a nonlinear dataset (circles and spirals), and handwriting recognition, showcasing the potential for on-chip computing. Nevertheless, multiplexed information processing is not feasible with current diffractive neural network devices, similar to conventional neural networks [85], [86], [87]. Furthermore, they typically have bulky light sources and detectors that do not allow them to combine the advantages of all-optical computation with sophisticated image sensor chips for optical wavelength-band image processing. Luo et al. demonstrated a polarized multiplexed metasurface-based all-optical neural network to accomplish a variety of recognition tasks, including the identification of fashion items and handwritten numerals, as shown in Figure 5b [31]. A CMOS imaging sensor is integrated with the physical network, facilitating the portability and miniaturization of an integrated sensing and computer chip. The illumination source for previous diffraction methods was monochromatic coherent light. In addition, Luo et al. reported the design of a broadband diffractive optical neural network capable of processing continuous wavelengths produced by temporally incoherent broadband light sources, performing specific tasks obtained through deep learning in a full-optics manner (Figure 5c) [88]. The results presented demonstrate that the D2NN framework is adaptable to broadband sources and capable of processing optical waves across a continuous and wide frequency range. Additionally, the computational power of D2NN tasked with machine learning can be substantially enhanced through multi-wavelength operation, made possible by the broadband diffractive network approach. The design methodology outlined here is not confined to THz wavelengths but can be extended to other regions of the electromagnetic spectrum, including the visible range. This advancement marks an important step in broadening the potential applications of diffractive optical neural networks, especially in scenarios where broadband functionality is crucial. Bai et al. introduced a pyramid diffractive network architecture that is specifically designed for unidirectional image magnification and demagnification in Figure 5d [89]. By restricting its possible solution space to a predefined bounded region based on the behavior of ray optics, the pyramid diffractive network architecture learns image scaling operations in one direction more efficiently than traditional uniform-sized D2NN designs. In comparison to standard D2NN models, this enables the pyramid diffractive network architecture to converge to a more optimal solution with fewer diffractive degrees of freedom.

However, the majority of reported all-optical D2NNs concentrate on tasks like object recognition and image classification that are absent of interaction with the environment. In contrast, networks with the ability to make decisions and take control have not yet been developed. However, the ultimate objective of AI is to directly emulate the decision-making and control processes of the human brain from high-dimensional sensory input. Qin et al. have developed all-optical D2NNs that mimic human-like control and decision-making abilities through deep reinforcement learning. Employing a residual design, these networks enable the discovery of optimal control policies through interaction with the environment and are easily scalable to existing optical devices (Figure 5d). Three distinct types of classic games including car racing, super Mario Bros., and tic-tac-toe were used to confirm their superior performance [90]. This innovation marks a step toward integrating intelligent decision-making into optical systems, bridging the gap between sensing and autonomous control.

The nonlinear activation layer in neural networks breaks the linear transformation relationship between data across multiple layers, enabling the network to learn more complex patterns. To achieve faster processing speeds and lower energy consumption, D2NNs have garnered significant attention in recent years, leading to the development of various optical nonlinear activation devices. The introduction of optical nonlinearity in D2NNs or optical computing can be achieved through several approaches: the first approach involves using materials with intrinsic nonlinear properties; the second relies on the nonlinear relationship between system input and output; and the third leverages higher-order optical nonlinear effects [91], [92]. Despite these advancements, several challenges remain. Current D2NN devices often rely on monochromatic light sources and bulky setups, limiting their scalability and practicality for integrated applications. Future developments in broadband diffractive optical networks and multiplexed meta-devices are expected to address these constraints, enabling more versatile and compact designs. By combining the inherent speed and efficiency of optics with advanced learning algorithms, D2NNs hold the potential to revolutionize computational paradigms across nanophotonics and beyond.

## 5 Optical quantum computing

Classical computers, while highly versatile and efficient in general-purpose tasks, face fundamental limitations in addressing problems of exponential complexity. Challenges such as combinatorial optimization, large-scale quantum system simulations, and integer factorization expose inefficiencies inherent to classical architectures [93], [94]. Additionally, their reliance on the von Neumann architecture introduces bottlenecks in data transfer and parallelism, while constraints in energy consumption and heat dissipation become more pronounced as transistor miniaturization approaches physical limits. Classical systems also lack the inherent randomness necessary for cryptographic security and struggle to model nonlinear or highly complex systems, underscoring the need for alternative paradigms like quantum computing.

**Figure 6:**
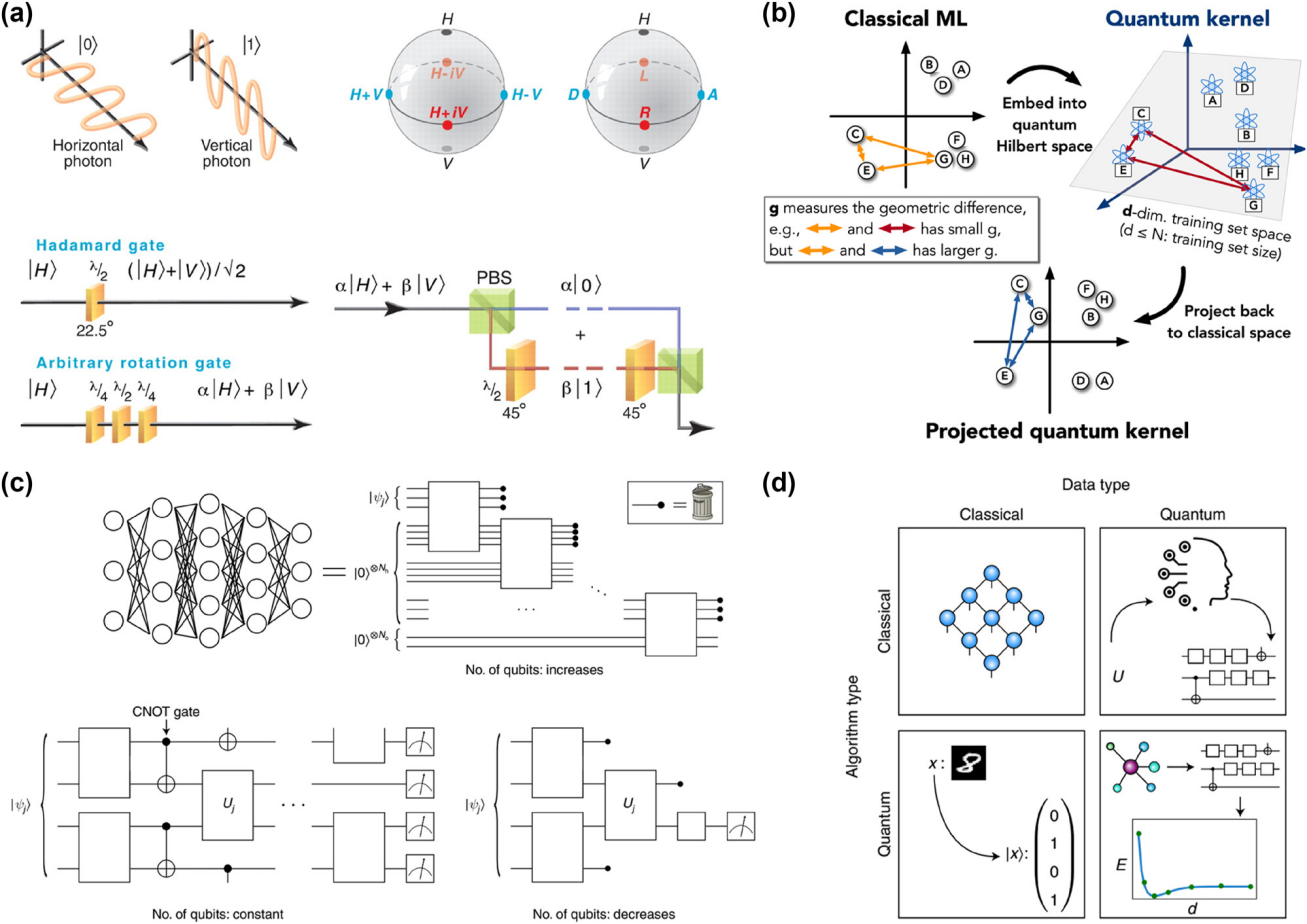
Optical quantum computing. (a) Single-photon qubits use horizontal photons for logical “0” and vertical photons for logical “1,” with states plotted on a Bloch sphere, controlled by birefringent wave plates, and converted between polarization and path encoding using a polarization beam splitter. Adapted from O’brieny et al. Science 318.5856 (2007): 1567–1570 [95]. Copyright 2007, American Association for the Advancement of Science. (b) Cartoon of the geometry (kernel function) defined by classical and quantum ML models. Adapted from Huang et al. Nature Communications 12.1 (2021): 2631 [96]. Copyright 2021, Nature. (c) A standard feedforward neural network with input, hidden, and output layers is an example of a QNN framework. Another alternative QNN strategy is to maintain the qubits fixed without replacing or discarding them, and QCNNs measure and discard qubits as the algorithm runs. Adapted from De Leon et al. Science 372.6539 (2021): eabb2823 [97]. Copyright, 2021, American Association for the Advancement of Science. (d) Paradigms of tasks that quantum machine learning can perform. Adapted from Cerezo, Marco, et al. Nature Computational Science 2.9 (2022): 567–576 [93]. Copyright 2022, Nature.

Optical quantum computing (OQC) offers a promising solution by leveraging photons as quantum information carriers (qubits). Photons exhibit unique properties such as high speed, low interaction with the environment, and immunity to decoherence, making them ideal for robust quantum operations. Qubits can exist in superpositions of quantum states, with polarizations (horizontal, vertical, or arbitrary) commonly representing the logical states $\left|0\right\rangle$ and $\left|1\right\rangle$ , as illustrated in Figure 6a [95]. This enables quantum parallelism, allowing photons to process multiple states simultaneously. OQC further supports diverse degrees of freedom for qubit encoding, including polarization, spatial paths, and time bins, providing flexibility and robustness in quantum operations. To elucidate the differences between classical and quantum machine learning (QML), Figure 6b compares their operational models [96]. In QML, data points {*xi*} (denoted as A, B, etc.) are embedded into high-dimensional quantum Hilbert spaces. Kernel functions, represented by arrows, measure similarities between data points, while the geometric differences *g* reflect variations in these measures between classical and quantum models. The effective dimensionality *d* of datasets in the quantum space illustrates the enhanced capacity of QML for complex data analysis. Additionally, Figure 6c highlights three distinct architectures of quantum neural networks (QNNs) [97]. The first model represents a dissipative QNN, which extends the concept of classical feedforward networks; in this setup, each node is associated with a qubit, and unitary operations connect qubits, with qubits being discarded after propagating information to the subsequent layer. The second model illustrates a conventional QNN, in which quantum data states traverse a quantum circuit without adding or discarding qubits in successive layers. Finally, the third model shows a convolutional QNN, where qubits are measured at each layer to reduce the data’s dimensionality while preserving its essential features. To date, QML encompasses a diverse range of tasks, as illustrated in Figure 6d [93]. These include classical applications, quantum-inspired algorithms, and quantum-specific optimizations. Quantum machine learning, for instance, can be used for quantum tasks like optimizing quantum experiments or finding quantum algorithms. QNNs can process both classical and quantum data. When using quantum-inspired techniques, even classical tasks can be categorized as QML. This perspective focuses primarily on QNNs, quantum kernels, and quantum deep learning, which constitute foundational areas within this rapidly evolving field. Despite significant advancements, O’Brien notes that substantial challenges must still be overcome to realize a large-scale optical quantum computer [95].

## 6 Advanced engineering applications

**Figure 7:**
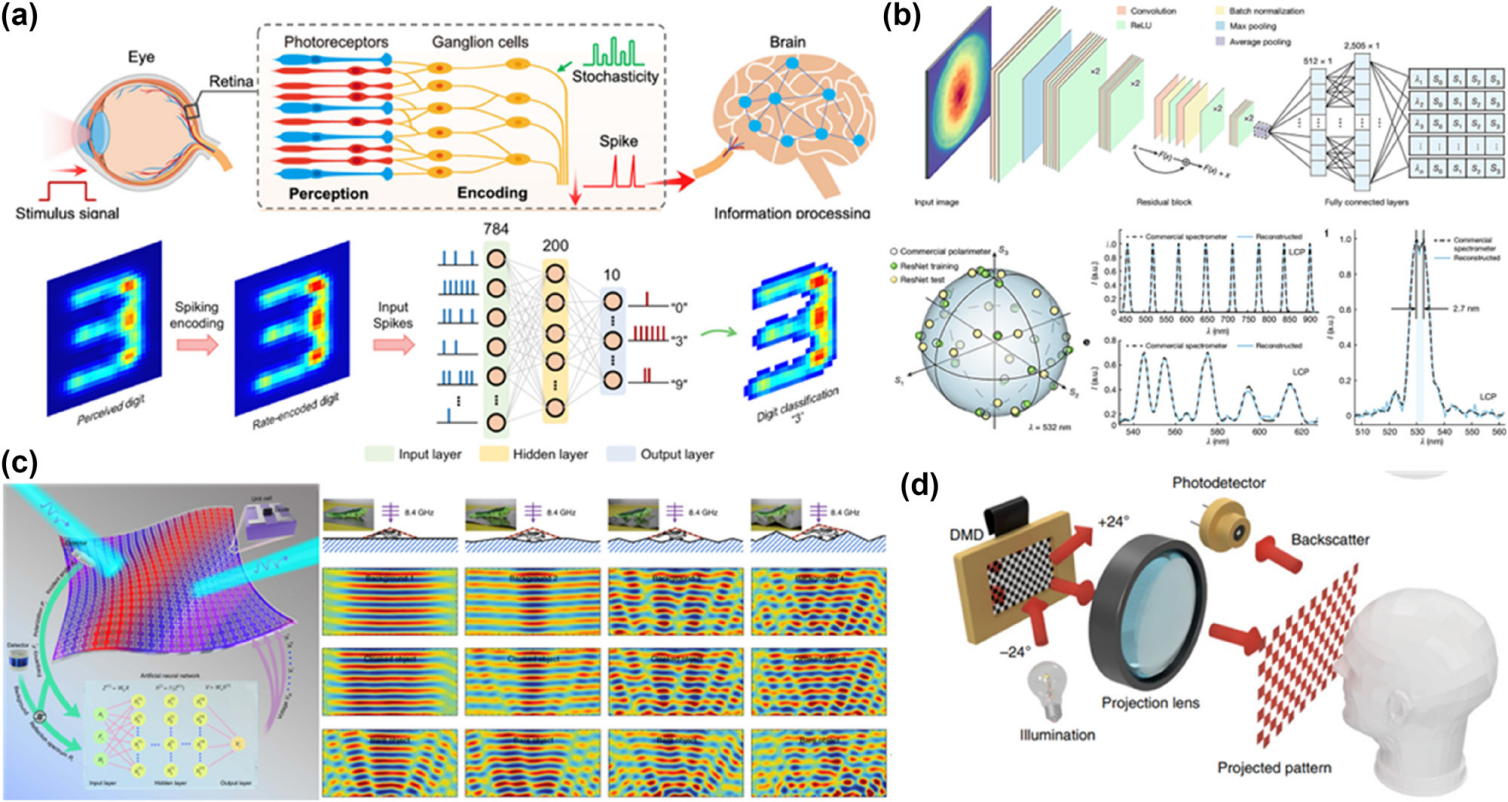
Artificial intelligence enabling advanced environmental interactive applications. (a) Schematic of the human visual system and the proposed 2D mid-IR optoelectronic retina. Adapted from Wang et al. Nature Communications 14.1 (2023): 1938 [109]. Copyright 2023, Nature. (b) Schematic of the modified ResNet-18 model and predicted results. Adapted from Fan et al. Nature (2024): 1–7 [110]. Copyright 2024, Nature. (c) Display of the selfadaptive cloak response to random backgrounds for a normal incident wave at 8.4 GHz. Adapted from Qian et al. Nature Photonics 14.6 (2020): 383–390 [111]. Copyright 2020, Nature. (d) Principles and prospects for single-pixel imaging. Adapted from Zhan et al. Optics Letters 47.11 (2022): 2838–2841 [112]. Copyright 2022, The Optical Society of America.

The integration of artificial intelligence (AI) into advanced engineering applications has become increasingly essential in the era of the Internet of Things (IoT) [98], [99], [100], [101], [102], [103], [104], [105], [106], [107], [108]. For the perception and identification of objects, infrared machine vision systems are crucial. In human vision, the retina’s photoreceptors (rods and cones) initially convert external stimuli into graded potentials, as depicted in Figure 7a. These graded potentials are then encoded into spike trains by ganglion cells, reflecting the inherent stochasticity of sensory transduction. Wang et al. applied spiking neural networks (SNNs) to encode and classify perceived images [109]. In their approach, mid-infrared (mid-IR) digit images are transformed into spike trains via rate encoding, which are subsequently processed by a trained fully connected SNN for digit classification. The predicted digit is determined by the output neuron that exhibits the highest spike rate. Simultaneous analysis of light’s intensity, polarization, and spectrum plays a pivotal role in applications like remote sensing, device miniaturization, chemical and biological characterization, optical communication, and astronomical observation. As shown in Figure 7b, Fan et al. demonstrated an advanced high-dimensional photodetection system capable of simultaneously characterizing wavelength and polarization information [110]. Their approach uses a single-shot measurement technique that combines a dispersive system to map spectral and polarization data with a deep neural network for decoding. This method delivers performance comparable to traditional polarimeters and spectrometers, enhancing the system’s efficiency in capturing complex optical data.

In the field of adaptive optics, the concept of an optimal invisibility cloak is particularly intriguing. An ideal cloak should dynamically adjust its internal configuration in response to external stimuli or changes in the surrounding environment, much like the adaptability of a chameleon. Intelligent, self-adaptive cloaks have high potential for real-time applications involving moving objects or complex, nonstationary environments. To address this, researchers have proposed an intelligent invisibility cloak powered by DL and realized using a tunable metasurface (Figure 7c) [111]. In this system, each metasurface element is independently controlled by a direct-current bias voltage applied to varactor diodes operating at microwave frequencies. A pretrained artificial neural network (ANN) computes and adjusts the bias voltages in milliseconds, allowing the cloak to autonomously adapt to dynamic incident waves and environmental changes.

Nowadays, high-level semantic sensing has been achieved using single-pixel sensing combined with an end-to-end neural network for joint optimization. However, this method can be computationally intensive, especially when sampling rates vary [112]. In reference [113], Zhan et al. present a weighted optimization approach for adaptive sampling single-pixel sensing [112]. This technique requires only a single network training session to handle dynamic sampling rates. A weighting scheme is introduced during the encoding process, which iteratively updates modulation patterns and their corresponding weights. The most effective modulation patterns, identified by the highest weights, are used for light modulation, significantly improving the efficiency of sensing in experimental applications [114].

**Figure 8:**
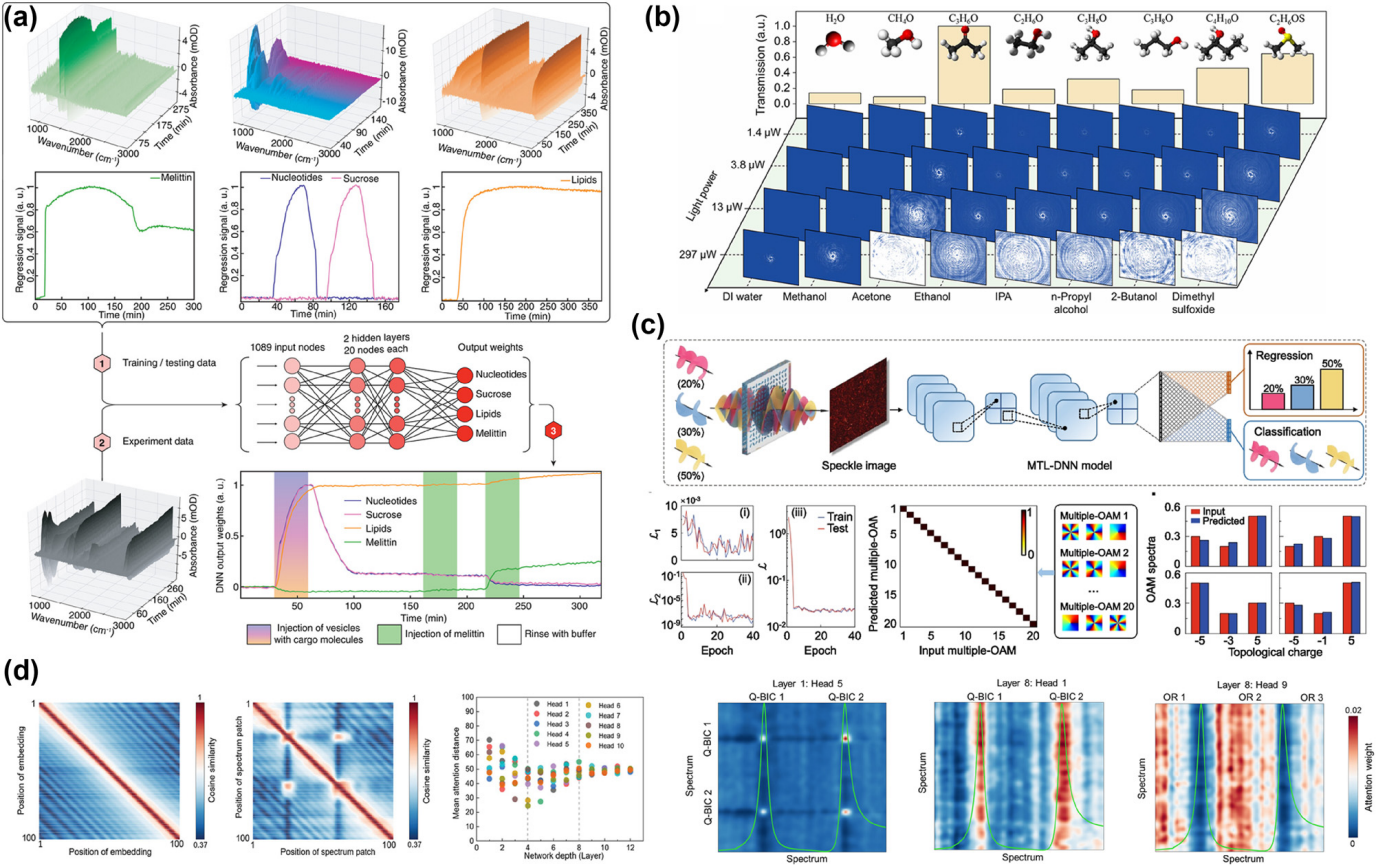
Artificial intelligence enabling advanced sensing applications. (a) A deep neural network to discriminate between different molecular components effectively. Adapted from John-Herpin et al. Advanced Materials 33.14 (2021): 2006054 [117]. Copyright 2021, Wiley. (b) Schematic of the *in situ* liquid identification process relying on metasurface-based vision intelligence. Adapted from Li et al. ACS Photonics 10.3 (2023): 780–789 [32]. Copyright 2023, American Chemical Society. (c) Intelligent multiple-OAM states sensing. Adapted from Zhu et al. PhotoniX 4.1 (2023): 26 [121]. Copyright 2023, Nature. (d) A framework for physically interpretable deep learning networks for bio-sensing. Adapted from Gao et al. Advanced Science (2024): 2405750 [43]. Copyright 2024, Wiley.

To increase our comprehension of both physiological and pathological biological processes, we must be able to identify and track biomolecules [115], [116]. It can be difficult to detect more than one or two target analytes, though, especially for processes where the net refractive index doesn’t vary much. This is where AI excels. John-Herpin et al. designed a D2NN that effectively differentiates between various molecular components, as shown in Figure 8a [117]. Large volumes of spectrotemporal data can be collected using the optofluidic method’s real-time format, which makes it quicker to construct a D2NN that can reliably distinguish between all significant classes of biomolecules [118], [119]. In Figure 8b, Li et al. demonstrated the potential of metasurface-integrated systems to simplify liquid chemical identification by leveraging unique vortex beam patterns and AI-powered classification, effectively bypassing bulky and complex spectrometric tools [120]. Figure 8c highlights the efficacy of the multi-task learning deep neural network (MTL-DNN) in detecting multiple orbital angular momentum (OAM) states and their power spectra [121]. A shared encoder and two task-specific heads make up the MTL-DNN architecture, which is used to classify OAM modes and regression of their power spectra. Speckle patterns generated by the disordered nematic liquid crystal (NLC) medium serve as inputs to the network. The confusion matrix demonstrates 100 % accuracy in identifying 20 distinct OAM states, encompassing various combinations of topological charges and power levels. These results demonstrate that the MTL-DNN achieves highly accurate recognition of various OAM states and their power spectra, underscoring the system’s capability for precise, high-dimensional light field sensing, and paving the way for advanced optical applications.

Notably, the black-box problem of neural networks has been a concern [122]. Gao et al. introduced the Metaformer model, emphasizing explainability in deep learning for metasurface sensor design by addressing the “black-box” limitations of conventional models [43]. This was achieved through spectral position encoding, preserving positional and spectral relationships by embedding critical patch information, as shown by cosine similarity analysis. Gao et al. further addressed this issue by introducing interpretable transformer networks. As shown in Figure 8d, the multi-head attention mechanism allows each head to focus on specific spectral features, such as Q-BIC resonance peaks or off-resonance regions [123], [124], [125]. Early network layers capture local features, while deeper layers integrate global patterns. This hierarchical learning enables the model to accurately predict high-Q spectral peaks and identify subtle interactions between resonance and off-resonance regions. Attention heatmaps illustrate how different layers and heads shift focus between local and global spectral details, highlighting the model’s ability to capture critical physical features essential for precise metasensor design predictions. In addition, some advanced applications, such as optical logic operations and imaging capabilities, have also attracted extensive attention [126], [127].

## 7 Insight and outlook

The integration of artificial intelligence in nanophotonics marks a significant leap forward in both fields, creating a synergy that leverages the strengths of each [58], [128], [129], [130], [131], [132], [133], [134]. AI-powered approaches have demonstrated remarkable capabilities in optimizing nanophotonic device designs, predicting complex behaviors, and accelerating the discovery of novel structures and configurations. These advancements are not only pushing the boundaries of what is possible in nanophotonics but are also facilitating the emergence of practical applications across diverse areas, like high-resolution imaging, advanced sensing, and quantum information processing. Looking ahead, the continuous evolution of AI technologies will further enhance our ability to solve intricate problems in nanophotonics. The development of more sophisticated AI models, along with improvements in computational power and data availability, will enable even more precise and efficient design processes. Additionally, the reciprocal influence of nanophotonics on AI, through innovations like optical computing and diffractive neural networks, promises to drive advancements in computational speed and energy efficiency.

However, despite these achievements, there are notable challenges that must be addressed. One major issue lies in the generation of large-scale, high-quality datasets essential for training AI models. Collecting and curating these datasets is often labor-intensive, particularly for highly specialized domains like nanophotonics, where experimental data can be scarce or expensive to obtain. Additionally, while AI models excel at specific tasks, their generalizability across diverse scenarios remains limited. This issue is compounded by the lack of interpretability in many machine learning algorithms, which can hinder their adoption in fields requiring high levels of reliability and interpretability. Another significant challenge is the computational cost associated with training sophisticated AI models, especially those incorporating physics-informed constraints or operating at high-dimensional parameter spaces. The energy consumption of such models presents sustainability concerns, which are particularly relevant in the era of green technology.

Future research will likely focus on the integration of AI with novel nanophotonic technologies, including metasurfaces, plasmonics, and quantum dots, exploring new paradigms for light manipulation and interaction at the nanoscale [135], [136], [137], [138], [139], [140], [141], [142], [143], [144], [145], [146], [147]. Moreover, interdisciplinary collaborations will be crucial in addressing the remaining challenges and exploiting the full potential of AI in nanophotonics [148], [149], [150], [151], [152]. Ultimately, the convergence of AI and nanophotonics will lead to the creation of next-generation optical devices with enhanced functionalities and unprecedented performance, transforming industries and opening up new frontiers in science and engineering [153], [154], [155], [156]. This dynamic interplay between AI and nanophotonics holds the promise of a future where intelligent, efficient, and highly integrated optical systems become a cornerstone of technological advancement (Table 1).

**Table 1:** Comparison between smart designs with AI and traditional designs.

	Smart designs with AI	Traditional designs
Design speed	High	Low
Innovation ability	High	Low
Design cycle	Short	Long
Optimization difficulty	Low	High
Degree of individuation	High	Ordinary
Accuracy	High	Relatively low
Data dependency	High	Weak
Interpretability	Relatively weak	Strong
Manufacturing constraint	High	Relatively low
